# Factors Associated With Cervical Cancer Screening Attendance in Hungary Based on the European Health Interview Survey

**DOI:** 10.3389/ijph.2024.1607509

**Published:** 2024-08-29

**Authors:** Jenifer Pataki, Gergő József Szőllősi, Attila Sárváry, Viktor Dombrádi

**Affiliations:** ^1^ Department of Integrative Health Sciences, Faculty of Health Sciences, University of Debrecen, Debrecen, Hungary; ^2^ Doctoral School of Health Sciences, University of Debrecen, Debrecen, Hungary; ^3^ Coordination Center of Research in Social Sciences, Faculty of Economics and Business, University of Debrecen, Debrecen, Hungary; ^4^ Patient Safety Department, Health Services Management Training Centre, Faculty of Health and Public Administration, Semmelweis University, Budapest, Hungary

**Keywords:** cancer control, cervical cancer, prevention, risk factor, screening

## Abstract

**Objectives:**

This study assessed the change in cervical cancer screening attendance across 10 years and identified the associated factors.

**Methods:**

Data from the European Health Interview Surveys in Hungary (2009, 2014, 2019) were analyzed with multivariate and multiple logistic regressions.

**Results:**

The analysis involved 4,850 participants, revealing a significant (*p* < 0.001) increase in screening attendance from 69% to 77% over 10 years. Factors significantly associated with higher attendance rates included a higher education level (tertiary level AOR = 2.51 [2.03–3.09]), being in a relationship (AOR = 1.59 [1.39–1.83]), the belief that one can do much for one’s health (OR = 1.26 [1.05–1.52]), and the absence of chronic health problems (AOR = 1.56 [1.33–1.84]). Lower screening odds were significantly correlated with worse self-perceived health status (AOR = 0.65 [0.52–0.81]) and less frequent doctor (AOR = 0.64 [0.54–0.76]) and specialist visits (AOR = 0.46 [0.39–0.53]).

**Conclusion:**

Enhancing cervical cancer screening rates requires tailored public health strategies, particularly targeting individuals with lower education and poor health perceptions. Public health initiatives and enhanced collaboration among healthcare professionals are required to further increase participation rates, particularly among the identified groups.

## Introduction

With 660,000 new cases in 2022, cervical cancer is the fourth most common cancer affecting women worldwide. Regional incidence variations are linked to disparities in immunization access, screening, and treatment facilities; risk factors such as HIV prevalence; and social and economic determinants including sex, gender bias, and poverty [1]. The majority of cervical cancers (99%) are associated with high-risk human papillomavirus (HPV) infection. HPV is a common virus, spread through sexual contact. The primary prevention steps should be immunization and health education [2–5]. Cervical cancer can be eradicated as a public health issue with a thorough prevention, screening, and treatment program [1–5]. Based on Global Cancer Observatory’s data, in Europe there were 58,219 new cervical cancer cases and 26,950 deaths in 2022 [6]. Hungary has 4.36 million women aged 15 years or older. Cervical cancer is the sixth most common cancer among Hungarian women overall and the third most common among those aged 15–44 years [7]. In 2022 the number of new cervical cancer cases was 964, and there were 482 deaths [6].

The purpose of cervical cancer screening is to identify precancerous changes in cervical cells, which can be treated to stop the development of cervical cancer. Cervical screening occasionally reveals malignancy. Treatment is typically less complicated when cervical cancer is detected early. Cervical cancer may have started to spread by the time symptoms appear, which makes treatment more challenging. Screening can be done in three major ways. First, an HPV test looks for high-risk HPV strains that can infect cells and cause cervical cancer. Second, the Pap test, also known as a Pap smear or cervical cytology, obtains cervical cells for examination for HPV-related changes that could develop into cancer if treatment is not received. Both precancerous and cervical cancer cells can be detected by it. Infections and inflammations are among the non-cancerous diseases that a Pap test might detect. Third, the HPV/Pap co-test checks for high-risk HPV and cervical cell abnormalities by combining the results of an HPV test and a Pap test. Several organizations, such as the American Cancer Society and the United States Preventive Services Task Force (USPSTF), have developed recommendations for cervical screening [8]. The USPSTF is a team of physicians and disease management experts who examine studies on the best ways to prevent diseases. It provides recommendations on how physicians may help prevent illnesses or detect them early [9]. The USPSTF advises Pap testing every 3 years from the ages of 21–29 years. For those aged 30–65 years, it recommends Pap testing every 3 years, an HPV/Pap co-test every 5 years, or an HPV test every 5 years [8, 10]. According to the updated cervical cancer screening guidelines of the American Cancer Society, HPV testing should begin at age 25 and continue every 5 years until age 65. However, testing every 3 years is still permissible with a Pap test or every 5 years with an HPV/Pap co-test [8, 11]. After the age of 65 years, the USPSTF suggests consultation with the healthcare provider about screening. Patients who have been screened regularly and have not had any problems usually do not need to be screened after the age of 65 years [8]. In its new guideline accepted in 2022, the Council of the European Union recommends using HPV testing for patients aged 30–65 years, with an interval of 5 years or more considering the individual cervical screening risk [12]. Even though that cervical cancer screening is essential for early detection of cervical cancer, there are several factors why women do not attend on screening regularly [13]. According to a meta-analysis there are barriers that have an impact on screening uptake, for example financial barriers, bureaucracy-related barriers, lack of trust, previous traumatic and negative care experiences or embarrassment about the examination [14]. Another study showed that the most frequent barriers are embarrassment, worrying from pain, having a bad experience in the past, difficulty having an appointment which would fit with commitments, being scared of what the test would find and intention to go for a test, but not getting round to it [15].

Since 2003, in Hungary, individuals between the ages of 25 and 65 years have had organized access to cervical cancer screening, based on Decree 51/1997 (XII.18) NM [16]. This Decree is about covering health services for the prevention and early detection of diseases under compulsory health insurance and confirmation of screening tests. Appendix 3 of the Decree contains a provision on targeted screening for public health purposes, according to which cervical screening is recommended every 3 years between the ages of 25 and 65 years, following a single negative screening test for public health purposes, with particular attention to cellular examination of cervical abnormalities (cytology) [17]. Screening is performed according to the method approved by the World Health Organization [18]. According to the National Health Insurance Fund Manager in Hungary, women who have not had a cervical screening in more than 3 years receive an invitation letter [16]. Regarding to the National Center for Public Health and Pharmacy, for organized screening, it recommends reviewing the target groups and screening methods and introducing new, but already pilot-tested, screening for public health purposes. In Hungary, breast screening, colorectal cancer screening and cervical cancer screening are currently the three major organized public health screening programs for which the aim is to increase participation [19]. To reduce cervical cancer incidence and screening attendance rate, there were different kind of initiatives in Hungary. The “Hungary National Health Strategy 2014–2020” one of its aims to improve reduction, early detection and treatment of cancer risk, which requires targeted interventions [20]. In addition, the Hungarian National Cancer Program notably addressing cancer prevention and control. It ensures that the screening procedure is reliable and high-quality by developing and implementing recommendations for cervical cancer screening [21]. Higher educational attainment might be linked to greater awareness and understanding of cancer prevention strategies, potentially leading to higher attendance rates in screening programs [22, 23].

In Hungary sampling for screening can be done by a gynaecologist or by the district midwife in the patient’s place of residence (known as a “screening midwife”), provided that the healthcare provider employing her has a license and a funding contract to carry out this specialized service [18, 24]. Furthermore, there are cervix self-screening tests (for example “Easy HPV Test”), which helps to screen for the presence of the HPV virus, so it can be helpful for early detection of cervical cancer [25, 26]. However, these tests are not yet widely known in Hungary. The primary prevention of cervical cancer is the HPV vaccine, which can prevent HPV from infecting the body by giving the immune system the ability to produce antibodies when the virus is present. In Hungary, since 2015 girls attending primary schools, and from 2020, boys attending primary schools have the opportunity to get the HPV vaccine free of charge, if their parents require it [18].

### Aims

Our study aimed to determine the change in cervical screening uptake prevalence among Hungarian women aged 25–65 years from 2009 to 2019 and identify possible influencing factors.

## Methods

### Database

Our data were obtained from the European Health Interview Surveys (EHISs), which were carried out in Hungary in 2009, 2014, and 2019 on representative samples using a standardized questionnaire. All three surveys were carried out under the supervision of Eurostat. The EHIS was conducted based on stratified two-step probability samples selected to produce precise estimates of health status indicators for the Hungarian population aged 15 years and older living in private households [27].

### Data

Our study’s main outcome was cervical screening participation. Because we focused on women between the ages of 25 and 65 years, those who did not meet these criteria were excluded from the analysis. Data was merged by aggregating those questions which are totally identical. The primary data collection year (2009/2014/2019) was represented by an indicator. The sociodemographic details of the respondents were recorded in the database, including their place of residence (city/village), marital status (have a partner/have no partner), highest level of education (primary/secondary/tertiary), and perceived income (good/poor). Responses to the query “How much can you do for your health?” Were also included (little/much). We also analyzed healthcare-related variables, such as the most recent visit with a doctor (within 12 months/more than 12 months) and a specialist (within 12 months/more than 12 months), satisfaction with the doctor (satisfied/not satisfied) and the specialist (satisfied/not satisfied), and the presence (have/not have) of a chronic health problem. The analysis also included smoking status (yes/no). To correct our analysis for potential confounders resulting from territorial heterogeneity, we lastly included a variable for the geographic region of residency based on the Nomenclature of Territorial Units for Statistics (NUTS2), comprising the planning and statistical regions of Hungary (Central Hungary/Southern Great Plain/Southern Transdanubia/Central Transdanubia/Western Transdanubia/Northern Great Plain/Northern Hungary). During the data cleaning process, only participants with complete data remained in the final sample. Therefore, those with incomplete data, who would not have contributed to the multiple analyses, were excluded (Supplementary file S1).

### Statistical methods

To assess the differences between those who attended cervical screening less than 3 years ago and those who did so more than 3 years ago, categorical features were analyzed using Pearson’s chi-square test. Consequently, two groups were created, those who participated screening within 3 years, and those who had not. The frequency distributions of the variables in the study were analyzed by these strata. Analyses of multivariate and multiple logistic regression were executed to identify variables potentially affecting the uptake of screening. Multiple logistic regression, using the enter method, was conducted with a binary variable representing screening participation within the last 3 years (1 = participated, 0 = did not participate). Predictor variables of 14, with the year of the survey, were included to assess their association with screening behavior. The results were expressed using *p*-values and adjusted odds ratios. The statistical analysis was performed using Stata Statistical Software (version 13.0, Stata Corp, College Station, TX, USA), and significance was defined as *p* < 0.05. The goodness of fit of the logistic regression models were tested with Hosmer-Lemeshow tests.

## Results

With 5,051 respondents from the 2009 dataset, 5,826 from the 2014 dataset, and 5,603 from the 2019 dataset, the initial sample size was 16,480. The 25–65 years age group included 1,809 women in 2009, 2,027 in 2014, and 1,804 in 2019. The final sample size was 4,850 after combining the three datasets (n = 5,640) and eliminating respondents (n = 790; 14%) who did not answer all the relevant research-related questions.

### Results of Chi-Square Tests Based On 2009, 2014, and 2019 Data

Education level showed a significant difference (*p* < 0.001) in all 3 years. The highest attendance rate was observed among those with tertiary education, followed by secondary, then primary (Table 1). In Concerning place of residence, significant results were only obtained in 2014 (*p* < 0.001), with those living in a city showing a higher participation rate. Marital status showed a similar significant difference (*p* < 0.001) in all 3 years, with residents who were married or partnered being more likely to undergo screening. Regarding perceived income, significant differences were observed in 2009 (*p* = 0.002) and 2014 and 2019 (*p* < 0.001): those with a good perceived income had a higher participation rate. Concerning self-perceived health status, we found significant differences in all years (*p* < 0.001), with respondents who said that their health status was good showing a higher attendance rate. The answer regarding how much someone can do for their health showed a significant difference (*p* < 0.001) in all 3 years; those who said that they could do much for their health had a higher participation rate. The existence of chronic health problems showed a significant result (*p* < 0.001) in 2014 and 2019; residents who had at least one chronic health problem were less likely to be screened than those who did not. Concerning the last meeting with a doctor, in 2009 (*p* < 0.001), 2014 (*p* < 0.001), and 2019 (*p* < 0.004), those who had visited their doctor within a year had a higher attendance rate. The last meeting with a specialist showed similar significant results (*p* < 0.001) in all 3 years. In 2014, respondents who said that they were satisfied with their doctor participated with significantly (*p* = 0.046) higher odds of cervical screening. Those who reported satisfaction with specialists demonstrated higher attendance rates, with statistical significance observed in 2009 (*p* = 0.039). Territorial heterogeneity was noticed in all 3 years, but no statistically verifiable differences were found.

**TABLE 1:** Results of chi-square tests based on 2009, 2014, and 2019 data of the European Health Interview Survey (Hungary, 2009, 2014, 2019).

Factors		2009 N = 1,643			2014 N = 1,819			2019 N = 1,388			All 3 years N = 4,850		
		Attended cervical screening in the past 3 years (N)	Attended cervical screening in the past 3 years (%)	*p*-value	Attended cervical screening in the past 3 years (N)	Attended cervical screening in the past 3 years (%)	*p*-value	Attended cervical screening in the past 3 years (N)	Attended cervical screening in the past 3 years (%)	*p*-value	Attended cervical screening in the past 3 years (N)	Attended cervical screening in the past 3 years (%)	*p*-value
Education level	Primary	429	60	<0.001^a^	471	65	<0.001^a^	122	57	<0.001^a^	1,022	61	<0.001^a^
	Secondary	429	72		499	81		593	77		1,521	77	
	Tertiary	268	82		400	84		352	86		1,020	84	
Residence	Urban	731	68	0.797	990	78	0.001^a^	720	78	0.158	2,441	75	0.007^a^
	Rural	395	69		380	70		347	75		1,122	71	
Marital status	Married or partnered	728	73	<0.001^a^	812	78	0.001^a^	788	80	<0.001^a^	2,328	77	<0.001^a^
	Not partnered	398	61		558	72		279	68		1,235	67	
Self-perceived income	Good	795	71	0.002^a^	1,111	79	<0.001^a^	959	79	<0.001^a^	2,865	77	<0.001^a^
	Poor	331	63		259	64		108	60		698	63	
Self-perceived health status	Good	987	70	<0.001^a^	1,285	77	<0.001^a^	990	79	<0.001^a^	3,262	75	<0.001^a^
	Poor	139	59		85	54		77	61		301	58	
How much can you do for your health?	Much	916	72	<0.001^a^	1,177	77	<0.001^a^	949	79	<0.001^a^	3,042	76	<0.001^a^
	Little	210	57		193	65		118	65		521	61	
Chronic health problem	At least one	823	68	0.502	595	71	<0.001^a^	504	72	<0.001^a^	1,922	70	<0.001^a^
	None	303	70		775	79		563	81		1,641	78	
Last meeting with doctor	<12 months	923	70	0.001^a^	1,131	77	<0.001^a^	884	78	0.004^a^	2,938	75	<0.001^a^
	≥12 months	203	61		239	68		183	70		625	66	
Last meeting with specialist	<12 months	850	75	<0.001^a^	1,033	79	<0.001^a^	790	80	<0.001^a^	2,673	78	<0.001^a^
	≥12 months	276	54		337	65		277	70		890	63	
Smoker	Yes	326	63	0.003^a^	349	69	<0.001^a^	283	70	<0.001^a^	958	67	<0.001^a^
	No	800	71		1,021	78		784	80		2,605	76	
How satisfied are you with your doctor?	Satisfied	914	69	0.666	1,176	76	0.046^a^	845	77	0.532	2,935	74	0.081
	Not satisfied	212	68		194	71		222	76		628	71	
How satisfied are you with your specialist?	Satisfied	707	70	0.039^a^	908	75	0.280	576	75	0.148	2,191	73	0.860
	Not satisfied	419	66		462	77		491	79		1,372	74	
Region (NUTS2)	Central Hungary	278	69	0.797	339	77	0.270	294	75	0.175	911	74	0.841
	Southern Great Plain	166	71		187	77		133	76		486	74	
	Southern Transdanubia	98	67		152	77		114	83		364	76	
	Northern Great Plain	189	67		241	73		171	77		601	72	
	Northern Hungary	155	71		144	69		133	83		432	74	
	Central Transdanubia	134	69		162	75		124	75		420	73	
	Western Transdanubia	106	65		145	78		98	72		349	72	
Year of the survey	2009	-									1126	69	<0.001
	2014										1370	75	
	2019										1067	77	

^a^ Significant (*p* < 0.05) findings.

### Screening Attendance Rate Based On Merged Data

From 2009 to 2019, a gradual increase occurred (69%; n = 1126, 75%; n = 1,370, 77%; n = 1,067) in the number of women who participated in cervical cancer screening, showing a positive trend (*p* < 0.001; Table 1). Cervical screening attendance showed significant differences regarding education level (*p* < 0.001). When compared to people with primary (n = 1,022; 61%) or secondary education (n = 1,521; 77%), those with tertiary education had higher participation rates (n = 1,020; 84%). A significant difference (*p* = 0.007) existed in the uptake of screening between women from rural (n = 1,122; 71%) and urban (n = 2,441; 75%) areas, with urban residents showing greater attendance rates. Individuals who were married or partnered (n = 2,328; 77%) exhibited significantly greater participation rates (*p* < 0.001) than those without a partner (n = 1,235; 67%). A significant difference existed by perceived income: those who indicated a good perceived income (n = 2,865; 77%) were more likely to be screened than those with a poor perceived income (n = 698; 63%; *p* < 0.001). Self-perceived health status showed a significant difference (*p* < 0.001) as well, with individuals reporting good health showing higher attendance rates (n = 3,262; 75%) than those who said that their health was poor (n = 301; 58%). Greater participation rates were reported by those who felt more empowered to manage their health (n = 3,042; 76%) than residents who said that they could do little for their health (n = 521; 61%; *p* < 0.001). Lower attendance was linked to the existence of at least one chronic health problem (n = 1,922; 70%) compared to respondents who did not have a chronic health problem (n = 1,641; 78%; *p* < 0.001). The uptake of screening was higher among residents who had visited their doctor within a year (n = 2,938; 75%) compared to those who had not (n = 625; 66%; *p* < 0.001). The participation rate of people who had seen a specialist within a year was significantly higher (n = 2,673; 78%) than those who had seen a specialist more than a year ago (n = 890; 63%; *p* < 0.001). Non-smokers had a higher attendance rate (n = 2,605; 76%) than smokers (n = 958; 67%; *p* < 0.001). No significant differences existed in screening between people who were satisfied with the doctor (n = 2,935; 74%) and those who were not (n = 628; 71%; *p* = 0.081). Similar results were found between those who were satisfied with the specialist (n = 2,191; 73%) and those who were not (n = 1,372; 74%; *p* = 0.860). No significant territorial heterogeneity was observed based on the merged sample (*p* = 0.841). The attendance rates ranged between 72% and 76%.

### Results of Logistic Regression Models Based On 2009, 2014, and 2019 Data

The *p*-values from the goodness-of-fit tests for the logistic regression models were all above 0.05 for the years examined, specifically *p* = 0.313 in 2009, *p* = 0.182 in 2014, and *p* = 0.408 in 2019, indicating that the models were well-fitted. In all 3 years, people with secondary and tertiary education had higher odds of participating in cervical screening than those with primary education (Table 2). The type of residence showed a significant result only in 2009: rural residents had a lower chance of screening uptake than their urban counterparts. In 2009 and 2019, respondents who were married or partnered had significantly higher odds of screening than people who did not have a partner. Self-perceived health status only showed a significant result in 2014, with those reporting poor health showing a lower chance of screening uptake than those who reported good health. How much individuals believed they could do for their health was significant in 2009, with those who believed that they could do much showing higher odds of screening. Chronic health problems were a significant factor in 2014 and 2019: residents with at least one chronic health problem had a lower chance of screening. The last meetings with a doctor and specialist had an association with screening uptake in all 3 years. Respondents who had visited their doctor or specialist within a year were more likely to be screened. Satisfaction with the doctor showed no significant association with screening participation, whereas satisfaction with the specialist was significant in 2009. People who stated that they were not satisfied with their specialist had a lower chance of screening uptake. Smoking status had no significant impact on the odds of screening attendance. In 2019, residents from Northern Hungary had a significantly higher chance of screening than those in Central Hungary.

**TABLE 2:** Results of logistic regression model based on 2009, 2014, and 2019 data of the European Health Interview Survey (Hungary, 2009, 2014, 2019).

Factors		2009 N = 1,643			2014 N = 1,819			2019 N = 1,388			All 3 years N = 4,850		
		Adjusted odds ratio	95% confidence interval		Adjusted odds ratio	95% confidence interval		Adjusted odds ratio	95% confidence interval		Adjusted odds ratio	95% confidence interval	
Education level	Secondary/primary	1.71^a^	1.32	2.22	1.91^a^	1.45	2.51	1.95^a^	1.37	2.79	1.76^a^	1.50	2.07
	Tertiary/primary	2.73^a^	1.90	3.91	2.26^a^	1.61	3.09	3.05^a^	1.93	4.82	2.51^a^	2.03	3.09
Residence	Urban/rural	0.75^a^	0.59	0.96	1.20	0.93	1.54	0.95	0.71	1.26	0.97	0.84	1.12
Marital status	Married or partnered/Not partnered	1.88^a^	1.49	2.36	1.33^a^	1.06	1.67	1.74^a^	1.31	2.31	1.59^a^	1.39	1.83
Self-perceived income	Poor/good	1.15	0.89	1.49	0.82	0.62	1.08	0.63^a^	0.43	0.91	0.89	0.75	1.05
Self-perceived health status	Poor/good	0.72	0.51	1.01	0.46^a^	0.31	0.68	0.79	0.49	1.25	0.65^a^	0.52	0.81
How much can you do for your health?	Much/little	1.60^a^	1.21	2.13	0.97	0.71	1.33	1.17	0.79	1.74	1.26^a^	1.05	1.52
Chronic health problem	None/at least 1	1.28	0.96	1.70	1.62^a^	1.25	2.10	1.71^a^	1.26	2.33	1.56^a^	1.33	1.84
Last meeting with doctor	≥12 months/<12 months	0.73^a^	0.55	0.99	0.58^a^	0.43	0.78	0.58^a^	0.40	0.82	0.64^a^	0.54	0.76
Last meeting with specialist	≥12 months/<12 months	0.38^a^	0.29	0.48	0.44^a^	0.34	0.57	0.60^a^	0.44	0.82	0.46^a^	0.39	0.53
How satisfied are you with your doctor?	Not satisfied/satisfied	1.09	0.81	1.47	0.75	0.55	1.03	0.90	0.63	1.26	0.91	0.76	1.15
How satisfied are you with your specialist?	Not satisfied/satisfied	0.70^a^	0.55	0.89	1.23	0.87	1.45	1.18	0.84	1.50	0.99	0.98	1.31
Smoker	No/yes	1.09	0.85	1.39	1.12	0.87	1.45	1.12	0.84	1.50	1.13	0.97	1.31
Region (NUTS2)	Central Hungary	Reference											
	Southern Great Plain	1.18	0.81	1.73	1.19	0.80	1.78	1.03	0.67	1.60	1.16	0.92	1.46
	Southern Transdanubia	1.10	0.71	1.70	1.45	0.93	2.24	1.53	0.91	2.58	1.33^a^	1.03	1.73
	Northern Great Plain	1.07	0.75	1.53	0.98	0.69	1.41	1.02	0.67	1.53	1.05	0.85	1.29
	Northern Hungary	1.32	0.89	1.96	0.80	0.53	1.20	1.74^a^	1.06	2.86	1.18	0.93	1.50
	Central Transdanubia	0.97	0.65	1.44	1.03	0.69	1.54	1.06	0.68	1.65	1.03	0.81	1.30
	Western Transdanubia	0.88	0.58	1.32	1.35	0.87	2.11	0.93	0.55	1.75	1.03	0.80	1.32
Year of the survey	2014/2009	-									1.15	0.98	1.35
	2019/2009										1.02	0.85	1.35

^a^ Significant (p < 0.05) findings.

### Results of Logistic Regression Models Based On Merged Data

The goodness of fit test showed a non-significant result in the case of merged data (*p* = 0.218), which suggest that the model based on the merged data was well-fitted. From 2009 to 2014 (AOR = 1.15 [0.98–1.35]) and from 2009 to 2019 (AOR = 1.02 [0.85–1.35]), the odds of cervical cancer screening participation did not show a significant result. Both secondary (AOR = 1.76 [1.50–2.07]) and tertiary education (AOR = 2.51 [2.03–2.07]) presented as protective factors; those with these education levels had significantly higher chances of screening compared to those with only primary education (Table 2). Place of residence did not show a significant correlation with screening uptake (AOR = 0.97 [0.84–1.12]). Respondents who were married or partnered had significantly higher odds of screening than those who did not have a partner (AOR = 1.59 [1.39–1.83]). No significant association was found regarding self-perceived income (AOR = 0.89 [0.75–1.05]). Worse self-perceived health significantly decreased the chance of participating in screening (AOR = 0.65 [0.52–0.81]). The belief in how much one can do for one’s health demonstrated a correlation with screening uptake. Residents who stated that they could do much for their health had higher odds (AOR = 1.26 [1.05–1.52]) of screening. The existence of at least one chronic health problem showed a significant result (AOR = 1.56 [1.33–1.84]). Meeting with the doctor (AOR = 0.64 [0.54–0.76]) and specialist (AOR = 0.46 [0.39–0.53]) beyond 12 months ago correlated negatively with screening participation. No significant results were found regarding satisfaction with the doctor (AOR = 0.91 [0.76–1.15]), satisfaction with the specialist (AOR = 0.99 [0.98–1.31]), or smoking status (AOR = 1.13 [0.97–1.31]). Territorial heterogeneity was observed regarding South Transdanubia: respondents from these regions had 33% higher odds (AOR = 1.33 [1.03–1.73]) of screening compared to residents from Central Hungary. No other significant territorial difference was found.

## Discussion

To reduce cervical cancer morbidity and mortality, screening is a crucial preventive public health measure for the early diagnosis of cervical malignancies. Attendance may have an impact on how successful screening programs are. Therefore, a proactive public health and health management stance is necessary. Knowledge of the subtle elements influencing cervical cancer screening participation is critical. According to our study the participation rate of screening in Hungary was 69% in 2009, 75% in 2014 and 77% in 2019. Attendance on cervical cancer screening varies in Hungary’s surrounding countries. Based on the Organisation for Economic Co-operation and Development (OECD) statistics, Slovakia’s program data indicates consistent rates between 2010 and 2022, ranging from 46%–48%. Romania has low program data rates, varying from 1.6% in 2013 to 4.5% in 2022, while survey data increased from 26.9% in 2017 to 38.9% in 2022. Slovenia’s program data was around 71%–74% and survey data had increased from 70.7% in 2017 to 80% in 2022. Finally, Austria maintained a high attendance percentage of 86.6% in 2014 and 84.6% in 2019, according to survey data [28]. This study may offer insight into the patterns of and factors influencing cervical cancer screening attendance between 2009 and 2019. Screening attendance rates increased from 2009 to 2019, indicating a potentially encouraging trend in Hungary. This may point to better access to healthcare, heightened public health knowledge and readiness, or strengthened educational initiatives during the research period. However, even with the general upward trend, disparities exist since variations in the attendance rates of different strata were noted. People with higher levels of education showed greater attendance rates than people with lower levels of education. This finding aligns with other studies [23, 29–33]. This may draw attention to the need for specialized treatments to overcome disparities in access to information about cervical cancer screening. Similar findings were made in a study that discovered a positive correlation of cervical cancer screening and HPV vaccination uptake with a high school or college education [34].

Differences existed in annual screening attendance between rural and urban locations, although these did not show a consistent trend of relevance over the 3 years. Urban residents were less likely than rural residents to be screened in 2009, according to multiple analyses – which is in line with the literature [35, 36] – even if a substantial difference in 2014 suggested higher participation rates. Researchers identified that women living in small settlements had lower odds of attending on cervical cancer screening than women from municipal towns [22]. Moreover, in a meta-analysis, researchers found that compared to patients from urban areas, those from rural areas had a higher likelihood of late-stage cervical cancer. The discrepancy can be explained by the fact that the place of residence affects the accessibility of healthcare services [37]. This pattern may point to obstacles in rural areas that require more research, such as inadequate healthcare facilities or awareness programs designed with rural residents in mind. Reducing these differences may help ensure that the advantages of cervical cancer screening are distributed more fairly throughout various geographical areas [38].

Marital status showed a significant association with screening attendance based on the 2009, 2014, 2019 and the merged data. Those respondents who were married or had a partner had higher odds of screening uptake. Other studies have also demonstrated that individuals who were married or partnered often showed a stronger propensity to attend cancer screenings, with their marital status a major predictor of attendance [22, 31, 39–43]. This emphasizes the importance of social aspects in public health programs, implying that social support is critical to healthcare decision-making.

Based on the univariate analysis conducted in all 3 years and the combined database, self-perceived income was a significant determinant of screening attendance. Respondents with greater income tended to have higher attendance rates, which aligns with other studies [44, 45]. On the other hand, the confounder-adjusted results showed that in 2019, higher participation in screening was linked to poorer self-perceived income. Even though Hungary’s public health authorities support free cervical cancer screening, notable differences may suggest that the relationship between perceived income and willingness to participate is dynamic and may be influenced by other factors.

Self-perceived health exhibited a significant outcome in 2014 and the merged data; those who reported that their health was poor were less likely to be screened for cervical cancer than those who said that their health was good. In 2009, the degree to which individuals believed that they could take responsibility for their health was a key determinant; individuals who felt highly capable of taking care of their health were more likely to undergo screening.

The importance of incorporating cervical cancer screening into the comprehensive care of people with chronic health conditions is highlighted by the lower attendance rates among these individuals. Their specific healthcare needs must be addressed. Based on our results more frequent meeting with the doctor and with the specialist showed higher participation rate on cervical cancer screening; those respondents who visited their doctor or their specialist within a year had higher odds of screening uptake. In a study, researchers found that frequently visiting health institutions was a significant factor in cervical cancer screening uptake [46]. Other studies have also suggested that less frequent contact with healthcare services negatively affects screening participation [47, 48]. Aras-Blanco et al found that in Europe, greater adherence to all preventive care services (such as cancer screening, influenza vaccination, and cardiometabolic screening) was associated with GP visits within a year [49]. The impact of the most recent appointment with a physician and specialist emphasizes the importance of prompt and routine healthcare interactions, even though satisfaction with healthcare providers did not always affect screening attendance. This highlights the possible role that medical practitioners play in encouraging cervical cancer screening and other preventive treatment during standard checkups. Satisfaction with the doctor and specialist showed no significant association with screening uptake in almost all cases. Significant result only emerged in 2009, people who said that they are not satisfied with their specialist had significantly lower odds on attending on cervical cancer screening. In case of smoking status, no significant association was found with screening participation. In another study researchers found similar results regarding smoking status [50], while others found that current smokers had lower chance of screening uptake compared to never smokers [51].

Based on univariate analysis regional differences existed, even though that the accessibility to screening is the same throughout Hungary. However, it should be noted that these differences were not statistically significant. According to the multivariate analysis Northern Hungary inhabitants having significantly higher chances of screening in 2019, and the analysis conducted on the merged data showed that respondents from Southern Transdanubia had higher odds of screening uptake. On that basis Hungarian researchers found territorial differences regarding cervical cancer screening attendance [52]. Based on our results further research on local elements influencing screening practices would be needed.

### Strengths and Limitations

This study used datasets from the EHIS, which offers a representative sample of the adult population of Hungary. Although the same methodology was used in all 3 years, an aggregated version of the data could be used for comparison purposes. Multiple logistic regression models allowed for the identification of significant determinants of screening uptake, offering insightful information for focused intervention techniques. However, importantly, the questionnaires were self-reported, so under-representation in the results is possible. Additionally, because of the methodological nature of the data collection, the database only included information on respondents, and no data was acquired on those who declined to participate. The primary strength of the study is the large sample size which followed appropriate sampling procedures. However, causal relationships could not be established. Furthermore, our research focused on individuals between 25 and 65, which is the target group that Hungary recommends for cervical cancer screening; whereas data from OECD statistics refers to individuals between 20 and 69. Therefore, caution is advised when comparing Hungary’s attendance rate to those of its surrounding countries. Finally, not all relevant socioeconomic data was available from the EHIS. For example, in 2021 a survey conducted among disadvantaged social groups showed that only 40.5% of Hungarian woman attended screening in the past 2 years [53], which is considerably lower than any of the subgroups investigated in this study.

### Conclusion

This research offers a thorough examination of Hungary’s cervical cancer screening attendance over 10 years. Achieving equitable access to cervical screening across varied groups requires tailored interventions that address educational and various socioeconomic characteristics. Based on our study the groups that need special attention are women with lower education level, who do not have a partner, who claim that their health is poor, who considers that they cannot do much for their health, who have at least one chronic health problem, and who meet with their doctor or specialist less than annually. To reach this aim, effective collaboration between healthcare providers and public health programs is essential. To increase screening participation and reduce health disparities, further research should examine the complex relationships between these variables and guide the creation of focused interventions.
