# Supplementary figures and images for: Factors Associated With Cervical Cancer Screening Attendance in Hungary Based on the European Health Interview Survey
Source: Int J Public Health. 2024 Aug 29;69:1607509. doi: 10.3389/ijph.2024.1607509 (PMC11390459; doi:10.3389/ijph.2024.1607509)

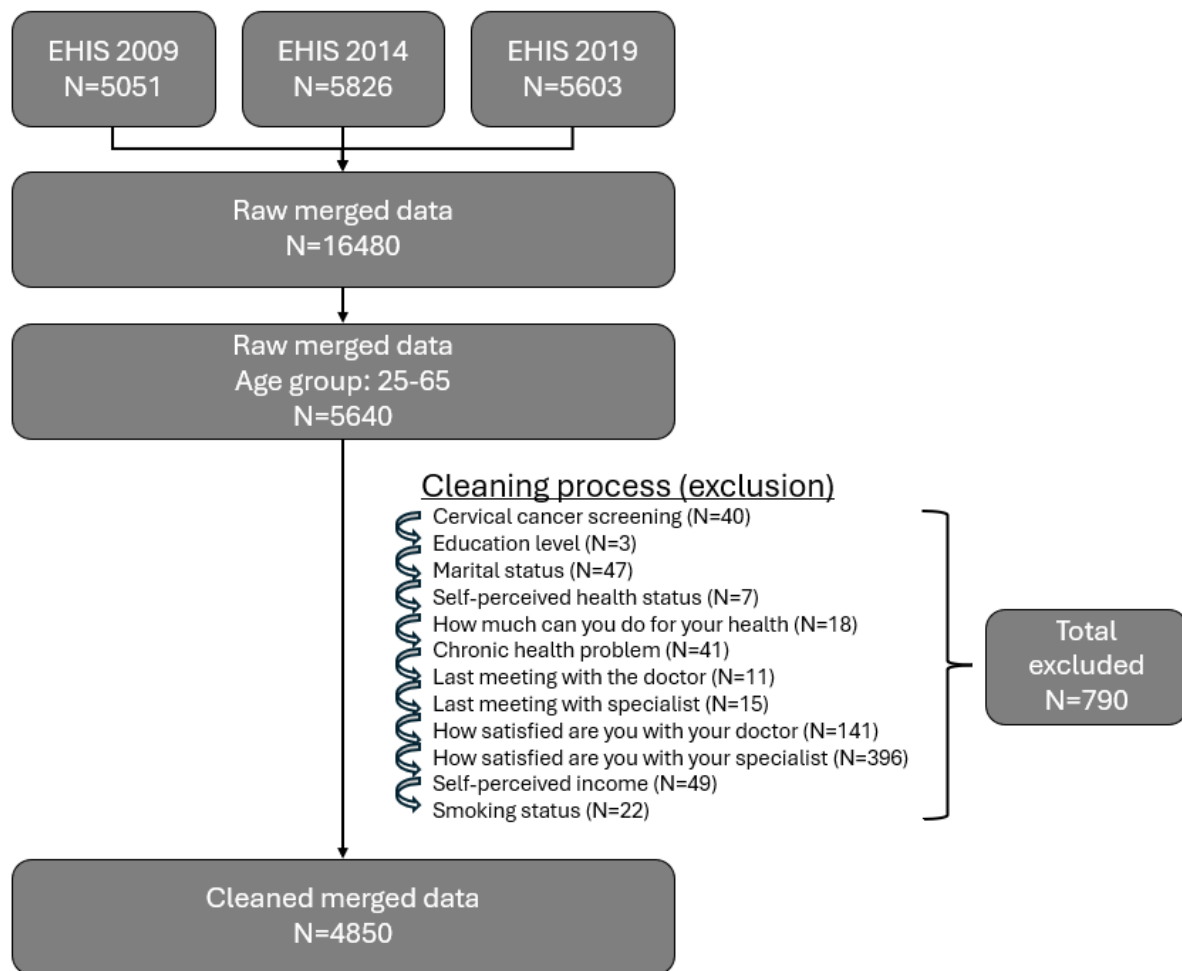

*Supplementary file 1: Data merging and cleaning process (Hungary, 2009, 2014, 2019)*

Supplement: Supplementary file 1 [file DataSheet1.PDF]
